# Design, Performance and Mechanisms of Asphalt Modified with Polyurethane and Hydroxylated Crumb Rubber

**DOI:** 10.3390/ma19081654

**Published:** 2026-04-21

**Authors:** Jun Xie, Junpeng Lin, Shaopeng Wu, Quantao Liu, Chao Li, Shibo Zhang, Huan Wang, Fusong Wang, Zoujun Wan

**Affiliations:** 1 State Key Laboratory of Silicate Materials for Architectures, Wuhan University of Technology, Wuhan 430070, China; xiejun3970@whut.edu.cn (J.X.); lljp@whut.edu.cn (J.L.);; 2 School of Safety Science and Emergency Management, Wuhan University of Technology, Wuhan 430070, China; 3 School of Materials Science and Engineering, Wuhan University of Technology, Wuhan 430070, China

**Keywords:** asphalt, polyurethane, hydroxylated crumb rubber, crosslinking reaction, modified asphalt, asphalt mixture

## Abstract

Under long-term heavy load and complex service environments, polyurethane-modified asphalt (PUMA) struggles to simultaneously satisfy the requirements of rutting and cracking resistance of asphalt pavements, as cyclic stress loading reduces the elastic recovery and low-temperature toughness of polyurethane (PU). To address this issue, this study employed hydroxylated crumb rubber (HCR), which is obtained by activating the surface of crumb rubber (CR) and can chemically crosslink with PU in asphalt to form a crosslinked network structure. The aim was to enhance the rutting and cracking resistance of PUMA by utilizing the elasticity and low-temperature toughness of CR. An orthogonal design was employed to systematically design a modified asphalt formulation with PU and HCR (PU/HCRMA) by controlling the isocyanate index and the contents of PU and HCR. The basic properties, rheological properties, and viscoelastic properties of PU/HCRMA were systematically investigated. The results demonstrate that the rutting and cracking resistance of PU/HCRMA are substantially enhanced, with an improvement of 28.91% in the rutting factor at 64 °C compared to PUMA and a reduction of 49.93 MPa in the stiffness modulus at −24 °C. Simultaneously, incorporating HCR in PUMA enhances its viscosity and flow resistance while reducing temperature susceptibility. Furthermore, by providing load-bearing sites, HCR endows PU/HCRMA with exceptional elastic recovery and deformation resistance. Results from FTIR and FM confirm the reaction between isocyanate groups in the PU prepolymer and the hydroxyl groups on the surface of HCR and the formation of HCR-PU crosslinked networks. Finally, PU/HCRMA asphalt mixtures demonstrate significant improvements in both rutting and cracking resistance. This research outcome provides a new direction for the development of high-performance road asphalt materials.

## 1. Introduction

Asphalt pavement, with its comprehensive advantages such as comfortable driving, has become the most dominant pavement structure type at present [1]. Under the conditions of long-term heavy load and increasingly complex service environments, polyurethane-modified asphalt (PUMA) struggles to simultaneously satisfy the performance requirements of rutting resistance and cracking resistance of asphalt pavements [2,3,4]. Cyclic stress loading progressively degrades the elastic recovery and low-temperature toughness of the polyurethane (PU) molecular chains, resulting in deterioration in the rutting resistance and cracking resistance of PUMA [5,6]. This deterioration accelerates the initiation and propagation of rutting and cracking in asphalt pavements, thereby shortening the service life of asphalt pavements. Zhang et al. discovered that once damage occurred, the modulus of thermosetting polyurethane-modified asphalt rapidly decreased, and the microcracks in the brittle network of thermosetting polyurethane were difficult to prevent or repair [7]. In addition, the raw materials of PU are primarily derived from crude oil, which is a non-renewable resource [8]. Therefore, the development of environmentally friendly methods for enhancing both the rutting resistance and cracking resistance of PUMA is of significant engineering importance and practical urgency.

Crumb rubber (CR), produced by shredding waste tires, is widely available and exhibits excellent elasticity [9,10,11]. When incorporated into asphalt, CR swells and partially interacts with asphalt. This process can impart enhanced elastic recovery and low-temperature toughness to asphalt binders [12,13,14]. Wu et al. [15] found that adding 22% and 28% CR enhanced the high-temperature performance and improved the low-temperature PG from −34 °C to −40 °C. In addition, activating the surface of CR can enhance its compatibility with asphalt, enabling it to better blend into asphalt, thereby resulting in asphalt with even better performance [16,17]. Kocevski et al. [18] achieved surface modification of CR by grafting with acrylic acid. Compared with unmodified CR, the modified CR improved the failure temperature of asphalt. Tian et al. [19] used sodium hydroxide and hydrogen peroxide to treat the surface of CR to enhance the compatibility and adhesion between CR and asphalt, enabling CR to maintain a favorable crosslinking network in the asphalt and providing interfacial rigidity and elastic resistance. Moreover, the composite modification of asphalt with CR and other modifiers can leverage their respective advantages to synergistically improve the various properties of asphalt [20,21]. Based on PG technology, Zhang et al. [22] compared the performance of a single SBS-modified asphalt mixture with that of SBS/rubber composite-modified asphalt mixtures. The results indicated that the high- and low-temperature properties of SBS/rubber composite-modified asphalt were improved.

Therefore, in this study, hydroxylated crumb rubber (HCR) was obtained by activating the surface of CR with sodium hypochlorite, then chemically crosslinking it with PU in asphalt to produce a polyurethane and hydroxylated crumb rubber composite-modified asphalt (PU/HCRMA). This approach leverages the elasticity and toughness of CR to enhance the rutting resistance and cracking resistance of PUMA while improving the compatibility between CR and asphalt. An orthogonal experimental design was employed to optimize the formulation of PU/HCRMA. A comprehensive evaluation of the basic properties, rheological properties, and viscoelastic properties of PU/HCRMA was conducted. Subsequently, Fourier transform infrared spectroscopy (FTIR) and fluorescence microscopy (FM) were used to characterize the structure and underlying mechanisms of PU/HCRMA. Finally, the rutting resistance and cracking resistance of PU/HCRMA in asphalt mixtures were evaluated.

## 2. Materials and Methods

**Figure 1:**
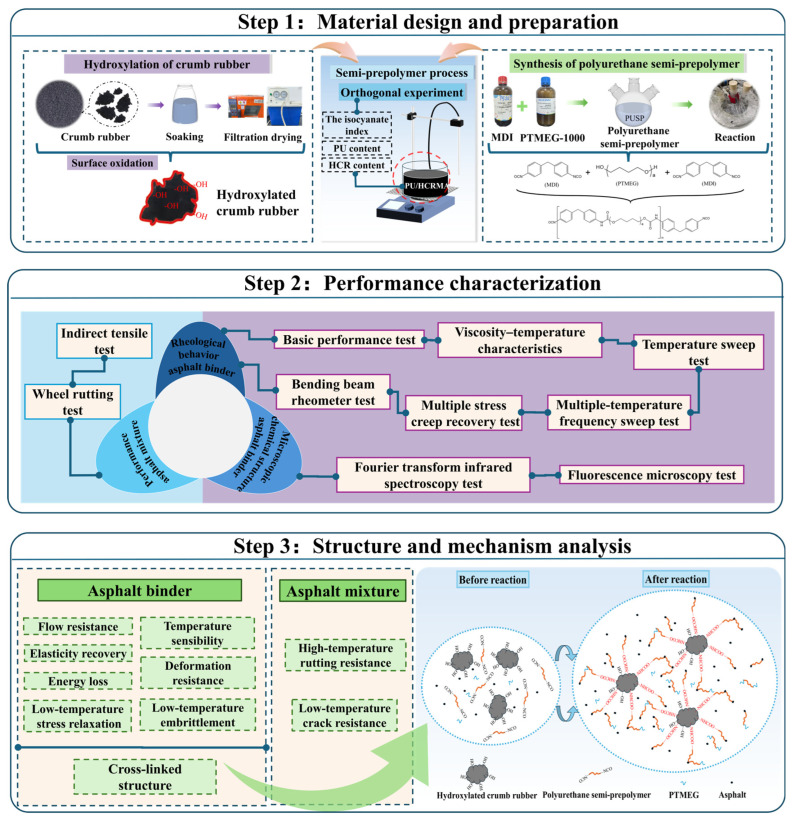
The technical roadmap of this study.

The technical roadmap of this study is presented below, the research having been conducted in three sections: (1) material design and preparation, (2) performance characterization, and (3) structure and mechanism analysis. Finally, the associated asphalt mixtures were investigated by wheel rutting and indirect tensile tests. The technical roadmap of this study is shown in Figure 1.

### 2.1. Materials

4,4′-Methylene diphenylmethane diisocyanate (MDI), dibutyltin dilaurate (DY-12) and poly (tetramethylene ether glycol) 1000 (PTMEG-1000) were purchased from Aladdin Biochemical Technology Co., Ltd. (Shanghai, China) for the synthesis of PU. DY-12 served as a common catalyst for polyurethane synthesis. The low-toxicity aromatic rings of MDI imparted rigidity and thermal stability, while the flexible ether linkages of PTMEG conferred low-temperature flexibility and hydrolysis resistance [3,23]. Their fundamental properties are listed in Table 1. A 40-mesh CR was supplied by Hubei Hanzhong Renewable Resources Co., Ltd., based in Xiaogan, Hubei, China, with its basic properties listed in Table 2. The sodium hypochlorite solution (chemically pure) was purchased from Sinopharm Chemical Practice Co., Ltd. (Shanghai, China). The base asphalt (BA) with a penetration grade of 60/80 was produced in Bohai, Shandong Province, with its fundamental properties listed in Table 3.

### 2.2. Synthesis of Polyurethane Semi-Prepolymer (PUSP)

According to the literature, there are three main processes for preparing PUMA: 1. the one-shot process; 2. the prepolymer process; 3. the semi-prepolymer process [22]. Among these, the semi-prepolymer process enables PU to disperse more effectively in asphalt. Therefore, this study employed the semi-prepolymer process to prepare PUMA. The process involved: (1) blending a portion of polyols into the asphalt, (2) adding PUSP for the reaction, and (3) the ultimate yield of PUMA.

In the process, PUSP synthesis was deeply affected by the isocyanate index. The typical synthesis procedure for PUSP with R = 1.5 proceeds as follows:

A three-necked flask was purged with nitrogen and maintained at 80 °C. Then, 4.10 g of MDI, 5.45 g of PTMEG-1000, and 0.10 g of DY-12 were introduced into the reactor. The mixture was mechanically stirred at 300 rpm to ensure homogeneity. The reaction was allowed to proceed at 80 °C for 2 h, after which the PUSP product was obtained.

### 2.3. Modification of Crumb Rubber with Sodium Hypochlorite

Studies have confirmed that hydroxyl groups form on the surface of rubber under the action of sodium hypochlorite [26]. To achieve the substitution of CR for chain extenders, this study employed sodium hypochlorite for the surface hydroxylation of CR, with the modification steps as follows:

First, the concentrated sodium hypochlorite solution was diluted with deionized water at a mass ratio of 1:3. Then, 400 g of CR was immersed in the diluted sodium hypochlorite solution and stirred thoroughly to ensure complete immersion. The mixture was then sealed and subjected to continuous stirring at 60 °C for 8 h. Finally, the HCR was collected by filtration, washing and drying.

### 2.4. Design and Preparation of Asphalt Modified with Polyurethane and Hydroxylated Crumb Rubber

#### 2.4.1. Designing Asphalt Modified with Polyurethane and Hydroxylated Crumb Rubber

This study investigated the modification effect of PU and HCR on asphalt through an orthogonal experimental design. Three key parameters that significantly influence the performance of asphalt were selected based on existing research: (1) the isocyanate index, (2) PU content, and (3) HCR content [27,28,29]. The levels for each parameter were determined according to prior engineering practice and research: (1) the isocyanate index ranged from 1.5 to 3.5, (2) the PU content from 3 wt% to 9 wt%, and (3) the HCR content from 3 wt% to 9 wt%. The orthogonal design is shown in Table 4.

Range analysis was employed on the orthogonal experimental data to identify the optimal formulation combination, as well as to determine the significance of each factor. $K_{i}$ denotes the sum of experimental results at the *i*-th level for a specific factor. ${\bar{K}}_{i}$ is the arithmetic mean of $K_{i}$. The comparison of ${\bar{K}}_{i}$ values revealed the impact trends of different levels within a single factor for performance, enabling identification of the optimal formulation combination according to specific requirements. Ranges were used to determine the significance of each factor’s impact on performance. In the range analysis, the range was highly positively correlated with the influence of the factor.

#### 2.4.2. Preparation of Asphalt Modified with Polyurethane and Hydroxylated Crumb Rubber

The PU and HCR composite-modified asphalt (PU/HCRMA) was prepared as follows:

First, a stainless-steel cup was filled with BA heated to 160 °C. Then, PTMEG-1000 and HCR were added, and the mixing paddle was rotated for 30 min. Subsequently, PUSP was added, and the mixing paddle was rotated for 30 min. During these periods, the mixing paddle speed was kept at 3000 rpm, and the temperature of the asphalt was kept at 160 °C. After that, fresh PU/HCRMA was obtained. Finally, PU/HCRMA was cured at 110 °C for 2 h to accelerate the reaction of isocyanate. In each of the subsequent asphalt performance tests, three parallel asphalt specimens were prepared, and a total of 351 asphalt specimens were tested.

### 2.5. Test Methods

#### 2.5.1. Basic Performance Tests

The penetration, ductility, softening point, and Brookfield viscosity of the asphalt were measured in accordance with Methods T 0604-2011, T 0605-2011, T 0606-2011, and T 0625-2011 of Standard JTG 3410-2025 [25], respectively.

#### 2.5.2. Storage Stability Tests

Poor compatibility between the modifier and the asphalt can reduce the road performance of modified asphalt. Therefore, it was necessary to evaluate the segregation of PU/HCRMA. The storage stability test for PU/HCRMA was conducted in accordance with the Standard JTG 3410-2025 [25]. Approximately 50 g of asphalt specimens was sealed in an aluminum tube and placed vertically in an oven at 163 °C for 48 h. The aluminum tube containing the asphalt was then transferred to a freezer at −20 °C for cooling for 4 h. The aluminum tube was longitudinally divided into three equal parts. Finally, the softening point test was conducted on the asphalt for the upper and lower sections. The softening point difference (*SPD*) was used to evaluate the compatibility between the polymer and the asphalt. The *SPD* is given in Equation (1).

(1) $$SPD=S_{bottom}-S_{top}$$

where SPD is the softening point difference (°C), $S_{bottom}$ is the softening point of asphalt in the bottom part, and $S_{top}$ is the softening point of asphalt in the top part.

#### 2.5.3. Viscosity–Temperature Characteristics Tests

The viscosity–temperature characteristics of the asphalt were determined using a Brookfield viscometer in accordance with Method T0625-2011 of Standard JTG 3410-2025 [25]. The flow activation energies of different asphalt specimens were determined by fitting the Arrhenius equation to evaluate temperature sensitivity and flow resistance [30]. The Arrhenius equation is given in Equations (2) and (3).

(2) $$\eta =Ae^{\frac{E_{\eta }}{\mathrm{RT}}}$$

(3) $$\ln\eta =\mathrm{lnA}+\frac{E_{\eta }}{R}\;\times \;\frac{1}{T}$$

where *η* is the viscosity of modified asphalt (Pa∙s); *A* is the regression coefficient; *R* is the universal gas constant, 8.314 J/(mol∙K); *T* is the absolute temperature (K); and $E_{\eta }$ is the flow activation energy (kJ/mol).

#### 2.5.4. Temperature Sweep Tests

The temperature susceptibility and rutting resistance of the different asphalt specimens were evaluated using a dynamic shear rheometer (DSR) instrument (Smartpave 102, Anton Paar, Graz, Austria). A temperature sweep test was performed on the asphalt in accordance with Method T 0628-2011 of Standard JTG 3410-2025 [25], employing a heating rate of 2 °C/min over the temperature range of 30 to 80 °C. The experiment utilized a strain-controlled mode with a 12% strain amplitude at an angular frequency of 10 rad/s. The complex modulus is one of the important indicators for assessing the overall deformation resistance of asphalt. The phase angle refers to the proportion of the viscous component and the elastic component in the asphalt.

#### 2.5.5. Multiple-Temperature Frequency Sweep Tests

The viscoelastic characteristics of asphalt binders exhibit significant temperature sensitivity and frequency dependence. To elucidate the rheological behavior of modified asphalt, multi-temperature frequency sweeps were performed. Multi-temperature frequency sweep tests were performed over a temperature range of 30 °C to 80 °C with linear frequency increments from 0.1 Hz to 10 Hz. Utilizing the time–temperature superposition principle, the Williams–Landel–Ferry equation, and the Huet–Sayegh model, the master curves of the complex modulus (G*), phase angle (*δ*), storage modulus and loss factor were constructed and fitted at the reference temperature from the obtained data. The storage modulus represents the elastic deformation performance of asphalt during the deformation process. The loss factor reflects the energy dissipation situation of asphalt under stress.

The model comprises two springs (denoted as $G_{0}$ and $G_{\infty }-G_{0}$) and two parabolic creep elements (denoted as *k* and *h*). This assembly of springs and dampers effectively simulates the linear viscoelastic behavior of asphalt across a broad range of frequencies and temperatures [31,32]. Notably, the model accurately reproduces the evolution of both the complex modulus (G*), the phase angle (*δ*), the storage modulus and the loss factor with respect to frequency.

The complex modulus of the Huet–Sayegh model is given in the following expression:

(4) $$G^{*}\left(\omega \right)\;={\;G}_{0}\;+\;\frac{G_{\infty }-G_{0}}{1\;+{\;\alpha (i\omega \tau )}^{-k}\;+\;{\left(i\omega \tau \right)}^{-h}}$$

where $G_{0}$ and $G_{\infty }$ represent the static and glassy modulus values (Pa), respectively, when *ω* tends to 0 and infinity; *k* and *h* represent the low- and high-frequency damping indices of the damper, respectively; *α* is a dimensionless constant; and *τ* is a characteristic time parameter related to temperature and the time–temperature transition equation. The value of *τ* only varies with temperature, and its evolution can be approximated by the Williams–Landel–Ferry equation obtained from Equations (5) and (6).

(5) $$\tau ={\;\alpha }_{T}\;\times {\;\tau }_{0}$$

(6) $$\log\left(\alpha_{T}\right)\;=\frac{-C_{1}(T-T_{\mathrm{ref}})}{C_{2}+(T-T_{\mathrm{ref}})}$$

where the characteristic time, $\tau_{0}$, is determined at the reference temperature; $\alpha_{T}$ is the displacement factor; $T_{\mathrm{ref}}$ is the reference temperature; and $C_{1}$ and $C_{2}$ are the fitting constants in the Williams–Landel–Ferry equation.

#### 2.5.6. Multiple-Stress Creep Recovery (MSCR) Tests

To quantitatively characterize the non-recoverable strain accumulation and elastic recovery performance of modified asphalt under multiple-stress repeated loading, the MSCR test was performed according to Method T 0647-2025 of Standard JTG 3410-2025 [25]. Dual stress levels (0.1 kPa and 3.2 kPa) were applied at 64 °C to quantify stress-dependent behavior. The recovery rate ($\varepsilon_{r}$), mean recovery rate ($R_{F}$), non-recoverable creep compliance ($J_{\mathrm{nr}}$), and its mean value ($J_{\mathrm{nrF}}$) were calculated using Equations (7)–(10):

(7) $$\varepsilon_{r}\left(F,N\right)\;=\;\frac{\varepsilon_{1}-\varepsilon_{10}}{\varepsilon_{1}}\;\times \;100$$

(8) $$R_{F}=\frac{\sum_{N=1}^{10}\varepsilon_{r}\left(F,N\right)}{10}$$

(9) $$J_{\mathrm{nr}}\left(F,N\right)=\frac{\min\left(\varepsilon_{1},\;\varepsilon_{10}\right)}{F}$$

(10) $$J_{\mathrm{nrF}}=\frac{\sum_{N=1}^{10}J_{\mathrm{nr}}\left(F,N\right)}{10}$$

where $\varepsilon_{r}\left(F,N\right)$, is the recovery rate (%) per cycle, $R_{F}$ is the mean recovery rate (%) across all cycles at a given stress level, $J_{\mathrm{nr}}\left(F,N\right)$ is the non-recoverable creep compliance (kPa^−1^) per cycle, $J_{\mathrm{nrF}}$ is the mean non-recoverable creep compliance (kPa^−1^) at a given stress level, $\varepsilon_{1}$ is the maximum strain during the loading phase (%), $\varepsilon_{10}$ is the non-recoverable strain at the end of the recovery phase (%), *F* is the applied stress (kPa), and *N* is the number of cycles.

#### 2.5.7. Bending Beam Rheometer (BBR) Tests

The low-temperature rheological properties of the asphalt specimens were evaluated using BBR tests performed according to Method T0627-2011 of Standard JTG 3410-2025 [25]. Beam specimens (127 mm × 6.35 mm × 12.7 mm) were conditioned at the target test temperature for 120 min. The creep stiffness (S-value) and creep rate (m-value) were subsequently measured at −6 °C, −12 °C, −18 °C, and −24 °C.

#### 2.5.8. Performance Tests of Asphalt Mixtures

**Figure 2:**
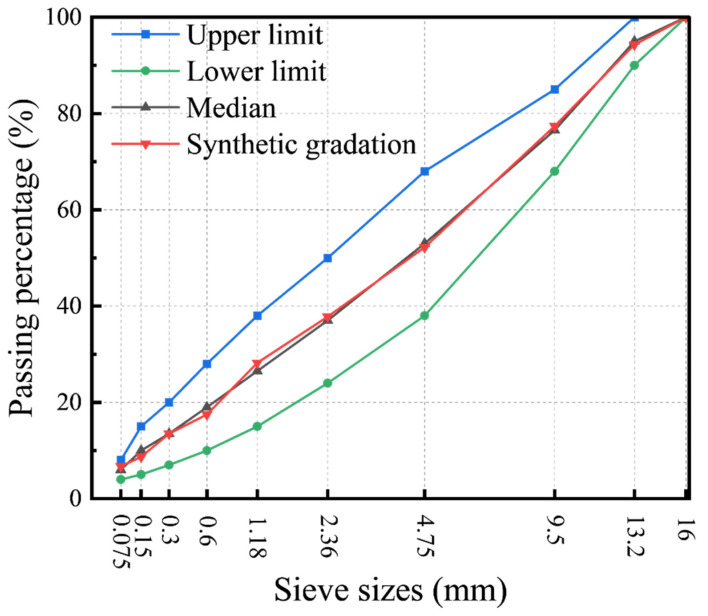
Gradation curve of asphalt mixtures.

To verify the actual performance of modified asphalt in road engineering, asphalt mixtures were designed, complying with Method T 0703-2025 of Standard JTG 3410-2025 [25]. The aggregate gradations of the asphalt mixtures were obtained by sieve analysis, and the gradation curve is shown in Figure 2. Subsequently, wheel rutting tests (T 0719-2025) and indirect tensile tests (T 0715-2025) were conducted to evaluate the rutting resistance and low-temperature cracking resistance, respectively. The wheel rutting tests were conducted at 60 °C under a load of 780 N, while the indirect tensile tests were performed at −10 °C at a loading rate of 50 mm/min. Three parallel asphalt mixture specimens were prepared for the wheel rutting tests and indirect tensile tests, and a total of 30 asphalt mixture specimens were tested.

#### 2.5.9. FTIR Tests

This study evaluated the modification effectiveness of CR on the surface and the reaction condition between HCR and MDI by characterizing the functional groups of CR, HCR, MDI, and the mixture of HCR and MDI. FTIR tests were performed over the spectral range of 4000 cm^−1^ to 400 cm^−1^ at a resolution of 4 cm^−1^. Triplicate measurements were performed for each specimen. Prior to the FTIR tests, both the CR and HCR specimens underwent rigorous drying to eliminate moisture interference and ensure spectral data accuracy.

#### 2.5.10. FM Tests

The dispersion of PU and HCR within the modified asphalt was characterized by FM imaging. Typically, asphalt appears light green or black in color, while polyurethane appears brighter and yellow or green in images, due to the absorption of aromatic hydrocarbons in asphalt [33].

## 3. Results and Discussion

### 3.1. Determination of the Formulation of PU/HCRMA

**Figure 3:**
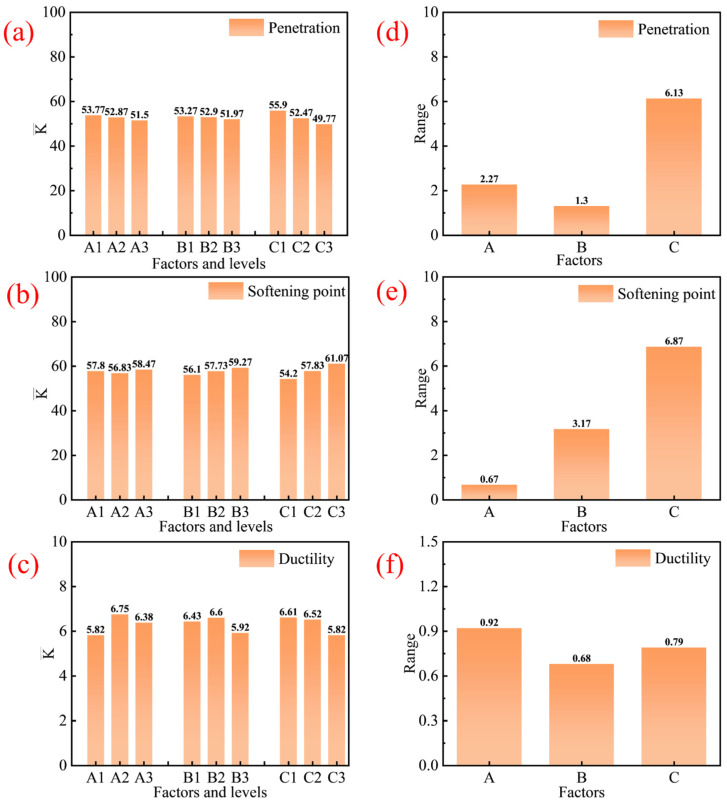
Results from the range analysis. (**a**) ${\bar{K}}_{i}$ values for penetration. (**b**) ${\bar{K}}_{i}$ values for softening point. (**c**) ${\bar{K}}_{i}$ values for ductility. (**d**) Ranges for penetration. (**e**) Ranges for softening point. (**f**) Ranges for ductility.

The significance of the isocyanate index, PU content, and HCR content on the performance of asphalt was evaluated using an orthogonal experimental design combined with range analysis, as depicted in Table 5 and Figure 3. The results demonstrate that the HCR content is the most significant factor affecting the penetration and softening point of asphalt, with ranges of 6.13 and 6.87, respectively. This indicates that HCR content plays a decisive role in enhancing the high-temperature deformation resistance of asphalt. This is attributed to HCR comprising elastic particles that impart superior elasticity to asphalt. Ductility is predominantly governed by the isocyanate index, as evidenced by its range of 0.92. This impact originates from dual mechanisms: (1) the isocyanate index regulates crosslinking density via chemical reactions between isocyanate and asphalt constituents and (2) the inherent rigidity of phenyl rings within isocyanate groups and the formation of hydrogen bonds impose steric constraints on molecular mobility [34]. The isocyanate index governs the structural ratio of hard segments to soft segments in polyurethanes, thereby balancing the performance at high and low temperatures. These synergistic effects collectively determine the temperature-dependent embrittlement behavior of asphalt binders.

As mentioned before, the comparison of ${\bar{K}}_{i}$ values reveals the impact trends of different levels of a single factor for performance, enabling identification of the optimal formulation combination. Lower ${\bar{K}}_{i}$ values in penetration indicate harder asphalt. Meanwhile, higher ${\bar{K}}_{i}$ values for softening point and ductility demonstrate enhanced high-temperature deformation resistance and improved low-temperature flexibility.

Therefore, based on the above analysis of the results, two modified asphalt formulations were selected: (1) An isocyanate index of 2.5, a PU content of 6%, and an HCR content of 3%. The asphalt prepared with this formulation was named PU/HCRMA-1 and exhibited good high-temperature rutting resistance and low-temperature cracking resistance. (2) An isocyanate index of 3.5, a PU content of 9%, and an HCR content of 9%. The asphalt prepared with this formulation was named PU/HCRMA-2. It possessed a relatively extreme high-temperature rutting resistance, although it had a relatively lower low-temperature cracking resistance. The aim of this formulation was to explore the enhancement effect on the high-temperature rutting resistance of PU/HCRMA. The formulations of the selected modified asphalts are shown in Table 6.

### 3.2. Basic Performance

**Figure 4:**
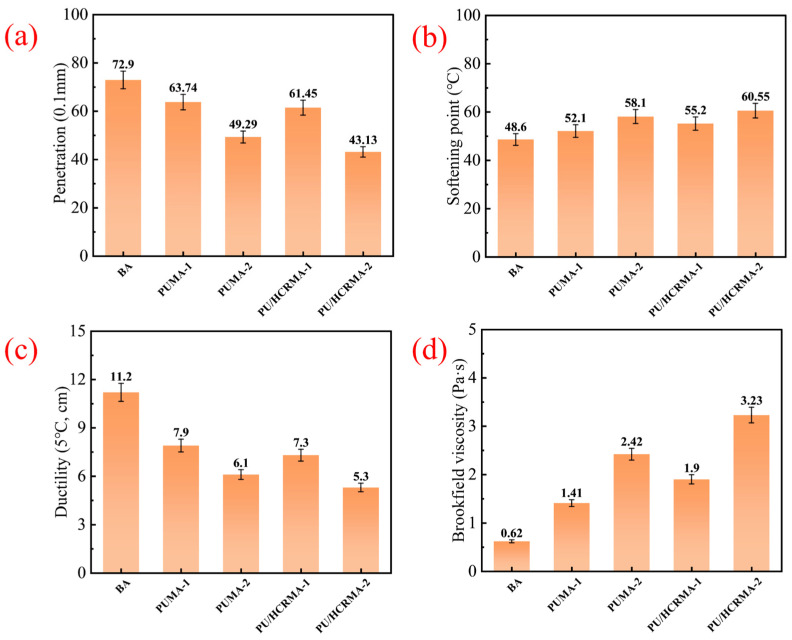
The results of basic performance tests for different asphalt specimens. (**a**) Penetration. (**b**) Softening point. (**c**) Ductility. (**d**) Brookfield viscosity.

The basic performance measures of asphalt include penetration, softening point, ductility and Brookfield viscosity. The basic performance test results for various modified asphalt specimens are shown in Figure 4. The results indicate that PU/HCRMA exhibits significantly lower penetration and ductility, along with a higher softening point and viscosity. This demonstrates that, compared to the modification effect of PU alone, the synergistic effect of HCR and PU can more effectively enhance the high-temperature deformation resistance and viscosity of base asphalt. However, it simultaneously reduces the low-temperature ductility capability of asphalt. The benzene ring in the isocyanate group exhibits inherent rigidity, which increases the stiffness modulus of asphalt, thereby leading to the decrease in penetration and the increase in the softening point. Meanwhile, CR absorbs aromatic fractions from the asphalt, which also contributes to an increase in the stiffness modulus, thus reducing the flexibility of asphalt and resulting in a decline in ductility.

### 3.3. Storage Stability

The softening point difference test is an important method for evaluating the storage stability of modified asphalt. Table 7 presents the softening point difference test results for modified asphalt specimens. PU/HCRMA-1 exhibits the lowest softening point difference, indicating that it has the highest storage stability and good compatibility among PU, HCR, and asphalt. It is worth noting that the softening point difference of PU/HCRMA-2 is greater than that of PUMA-2, while that of PU/HCRMA-1 is smaller than that of PUMA-1. This is because the content of HCR in PU/HCRMA-2 is excessively high, preventing complete crosslinking with PU, which results in poor storage stability. Although the softening point differences of PU/HCRMA-2 and PUMA-2 are relatively high, they still meet the specification requirements for asphalt pavements. Therefore, all modified asphalts prepared in this study can be used for asphalt pavements.

### 3.4. Viscosity–Temperature Characteristics

**Figure 5:**
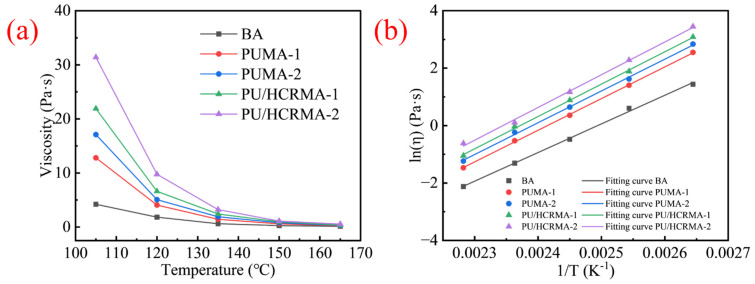
The results for viscosity–temperature characteristics. (**a**) Viscosity–temperature curves. (**b**) Arrhenius equation fitting curves.

Viscosity–temperature curve testing is essential to determine optimal mixing and compaction temperature. Figure 5a and Figure 5b respectively show the viscosity variation of modified asphalt specimens across different temperatures and the fitting curves for the Arrhenius equation. The results reveal that all asphalt specimens exhibit decreasing viscosity with rising temperature. The viscosity values of different asphalt specimens at 150 °C are very similar, which is attributed to asphalt approaching a Newtonian fluid at high temperature. Under this condition, asphalt molecular thermal motion intensifies, viscous flow components dominate, and the viscosity-enhancing effect of different asphalt modifiers is greatly weakened, resulting in the convergence of viscosity values among different asphalt specimens. Notably, PU/HCRMA consistently demonstrates higher viscosity than both base asphalt and PUMA at all tested temperatures. This is primarily attributed to chemical reactions among HCR, PU, and asphalt resulting in the formation of a crosslinked network that substantially increases viscosity. Detailed analysis of these chemical reactions and resultant structures will be presented in Section 3.10. Additionally, the incorporation of HCR inherently elevates the viscosity of asphalt due to physical swelling and further volumetric expansion [35]. HCR absorbs a certain proportion of aromatic fractions of asphalt, resulting in a volumetric expansion.

To quantitatively characterize the rheological properties of asphalt specimens at varying temperatures, flow activation energies were calculated using the Arrhenius equation. Table 8 demonstrates that all fitting correlation indices exceed 0.99, confirming that the Arrhenius equation accurately describes the flow activation energy of PUMA. Despite utilizing different modifiers, the rheological behavior of these asphalt variants still conforms to classical asphalt rheological models. The results shown in Figure 5 and Table 8 reveal that the specimens of two types of PU/HCRMA exhibit the highest flow activation energy, indicating that they have the strongest resistance to flow and thus exhibit the lowest temperature susceptibility.

### 3.5. Temperature Dependence of Rheological Performance

**Figure 6:**
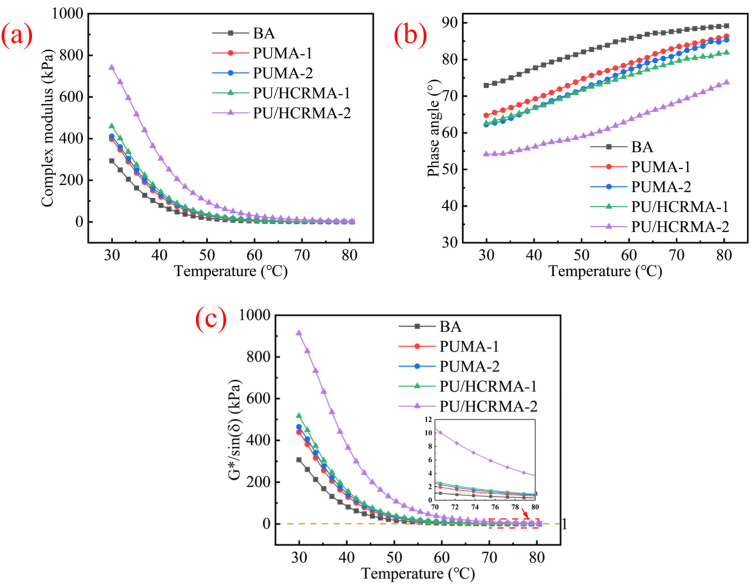
The results of temperature sweep tests. (**a**) Complex moduli. (**b**) Phase angles. (**c**) Rutting factors.

Figure 6a, Figure 6b and Figure 6c respectively illustrate how the complex modulus, phase angle, and rutting factor of all the asphalt specimens vary with temperature. As temperature increases, all specimens exhibit declining trends in both complex modulus and rutting factor (G*/sin(δ)), while the phase angle shows an upward trend. Owing to the accelerated relaxation of asphalt at elevated temperatures, viscous flow becomes the dominant response, thereby diminishing the rutting resistance of asphalt. At the same temperature, PU/HCRMA demonstrates the highest complex modulus and rutting factor along with the lowest phase angle. The rutting factor of PU/HCRMA at 64 °C increased by at least 28.91%. This indicates that PU/HCRMA possesses enhanced elastic properties and superior rutting resistance at high temperatures. The improvement primarily stems from the higher inherent elasticity of HCR. Furthermore, the physical swelling of HCR and its interfacial interaction with asphalt molecules collectively facilitate the formation of a network structure, thereby endowing the asphalt with enhanced elasticity. The elastic recovery capability of all asphalt specimens will be quantitatively characterized in Section 3.7 through MSCR tests.

### 3.6. Temperature–Frequency-Dependent Rheological Behavior

**Figure 7:**
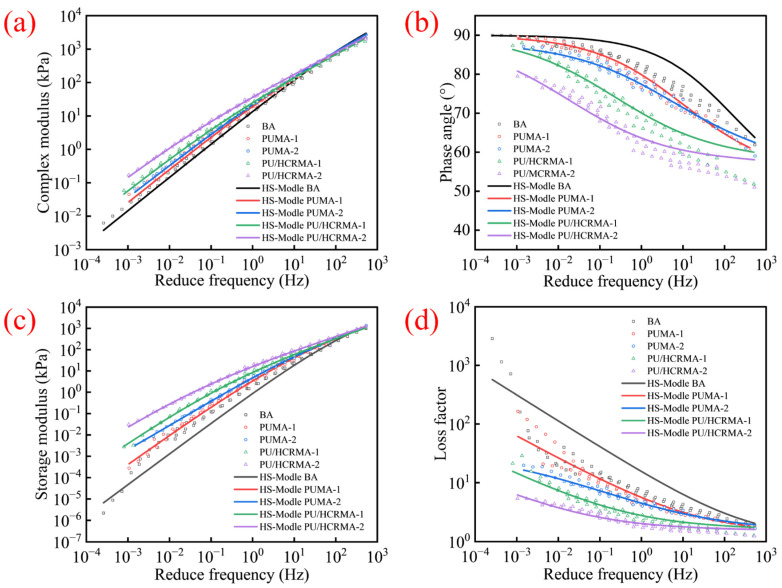
The results of master curves of different asphalt specimens. (**a**) Master curves of complex moduli. (**b**) Master curves of phase angles. (**c**) Master curves of storage moduli. (**d**) Master curves of loss factors.

The rheological properties of asphalt binders exhibit significant temperature–frequency dependence [36,37]. Figure 7 shows the results of the multiple-temperature frequency sweep tests, which give important information about complex moduli, phase angles, storage moduli and loss moduli. The main curve based on the time–temperature superposition principle and the Huet–Sayegh model (reference temperature: 50 °C) indicates that as the frequency increases, the complex moduli and the storage moduli of all specimens show a non-linear increase with a gradually slower growth rate (Figure 7a,c). The phase angle and the loss factor show a non-linear decrease with a gradually stabilizing decline, approaching a plateau zone for PU/HCRMA (Figure 7b,d). It is speculated that the storage modulus increases with the increase in CR in the binder. During the DSR frequency sweep, asphalt undergoes shear deformation, causing interactions between CR particles to profoundly influence the rheological properties of the asphalt binder. When the content of CR is high, the PU/HCRMA exhibits increasingly pronounced elastic characteristics in the mid-frequency range. This effect endows the PU/HCRMA with a more stable viscoelastic equilibrium state.

### 3.7. Resistance to Permanent Deformation and Recovery Capability

**Figure 8:**
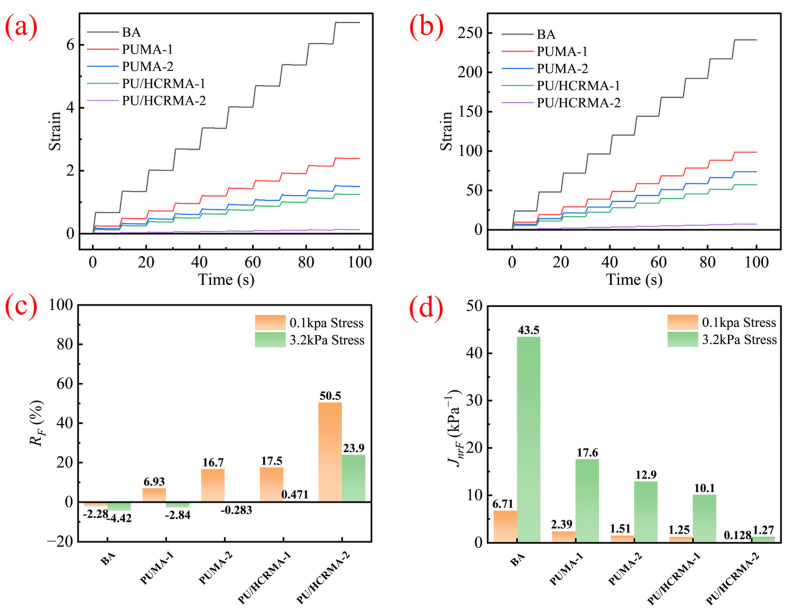
The results of MSCR tests of different asphalt specimens. (**a**) Strain of 0.1 kPa stress. (**b**) Strain of 3.2 kPa stress. (**c**) Mean recovery rate ($R_{F}$). (**d**) Mean non-recoverable creep compliance ($J_{\mathrm{nrF}}$).

The elastic recovery capability and permanent deformation characteristics of various asphalt binders under differential stress were quantitatively characterized through MSCR test. Figure 8a and Figure 8b display the deformation behavior of all asphalt specimens under stresses of 0.1 kPa and 3.2 kPa, respectively. In addition, Figure 8c and Figure 8d show the mean recovery rate ($R_{F}$) and mean non-recoverable creep compliance ($J_{\mathrm{nrF}}$) values for all asphalt specimens, respectively. The results indicate that under sustained stress loading, PU/HCRMA exhibits the lowest final strain, the highest recovery rate, and the lowest non-recoverable creep compliance. Based on quantitative calculations, PU/HCRMA achieves the highest recovery rate of 50.5% and the lowest non-recoverable creep compliance of 0.128 kPa^−1^, which values are significantly lower than those of PUMA and the base asphalt. Notably, under a stress of 3.2 kPa, the recovery rates of both PUMA and the base asphalt are negative, whereas PU/HCRMA exhibits a positive recovery rate, reaching up to 23.9%. This indicates that PU/HCRMA possesses outstanding elastic recovery and resistance to permanent deformation under heavy load conditions, effectively mitigating pavement rutting and fatigue damage. This is attributed to the superior elasticity of HCR, which enables it to act as an elastic energy storage unit capable of absorbing more momentum energy.

### 3.8. Low-Temperature Creep Stiffness and Relaxation Performance

**Figure 9:**
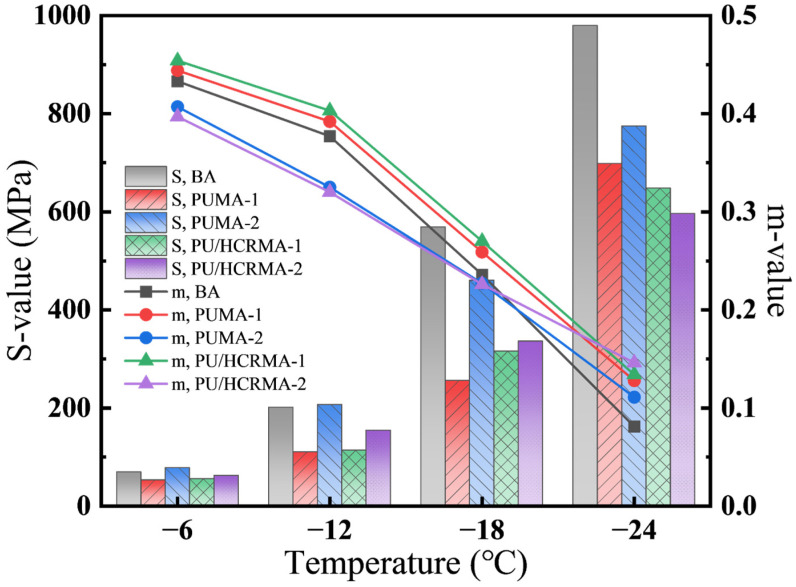
The results of the BBR test for asphalt specimens.

Figure 9 illustrates the stiffness moduli (S-values) and creep rates (m-values) for all asphalt specimens at various low temperatures. As the temperature decreases, the stiffness of all asphalt specimens increases while the creep rate declines. The occurrence of higher S-values accompanied by lower m-values indicates reduced stress relaxation capacity and increased embrittlement, signifying diminished low-temperature performance. This results from the reduced flow capacity of the asphalt molecules, whereby the timely release of stresses is hindered, consequently causing both elevated brittleness and diminished stress relaxation.

At −24 °C, PU/HCRMA exhibits superior low-temperature cracking resistance compared to PUMA, as evidenced by its lower stiffness modulus and higher creep rate. Its stiffness modulus decreased by at least 49.93 MPa. Research indicates that the low-temperature performance of PUMA is significantly influenced by the content and properties of the soft segments in the PU [38]. As shown in Figure 9, the S-value of the modified asphalt increases with the isocyanate index, indicating that an insufficient soft segment content in the modifier leads to reduced flexibility of the asphalt at low temperatures. Furthermore, PUMA incorporates PTMEG-1000 polyether polyol, which exhibits exceptional low-temperature flexibility [39]. Consequently, all PUMA specimens demonstrate significantly superior low-temperature toughness compared to base asphalt. In addition, HCR significantly enhanced the low-temperature cracking resistance of PU/HCRMA. Since HCR maintains effective low-temperature toughness below −24 °C, it improves the stress relaxation capability of PUMA, enabling PU/HCRMA to dissipate stress more rapidly under loading. This reduces the extent of low-temperature cracking, resulting in enhanced low-temperature cracking resistance. However, at higher HCR contents, the swelling of HCR particles absorbs excessive aromatic fractions of asphalt, resulting in increased brittleness and elevated moduli, ultimately compromising the low-temperature performance of PU/HCRMA. Thus, adding an appropriate amount of HCR enhances the low-temperature cracking resistance of PUMA.

### 3.9. Performance of Asphalt Mixtures at High and Low Temperatures

**Figure 10:**
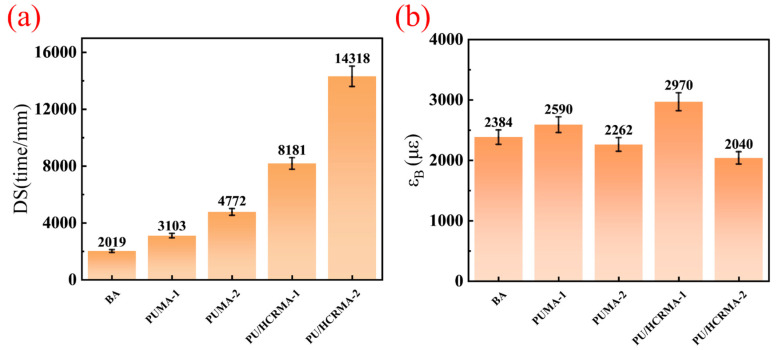
The performance of asphalt mixtures. (**a**) Wheel rutting test. (**b**) Indirect tensile test.

Wheel rutting tests and indirect tensile tests can effectively evaluate the high-temperature performance and low-temperature performance of different asphalt mixtures [40,41]. Figure 10a presents the results of the wheel rutting tests for asphalt mixtures prepared with various asphalt binders. By comparing their Dynamic Stability (DS), the high-temperature rutting resistance of the asphalt binders can be ranked in descending order as follows: PU/HCRMA, PUMA, and base asphalt (BA). The DS of the asphalt mixtures PU/HCRMA-2 and PU/HCRMA-1 reaches 14,318 time/mm and 8181 time/mm. This is attributed to the fact that the PU/HCRMA binder has the strongest ability to resist deformation and elastic recovery at high temperatures, while BA has a higher proportion of viscous components and poorer high-temperature performance. The results are consistent with the aforementioned tests and analysis.

Maximum tensile strain ($\varepsilon_{B}$) is a key indicator in the assessment of the low-temperature performance of asphalt mixtures. Indirect tensile test results (Figure 10b) show that the PU/HCRMA-1 asphalt mixture exhibits the highest low-temperature cracking resistance, with a maximum tensile strain of 2970 με. This result aligns with the BBR test results in Section 3.8, demonstrating the strongest stress relaxation and the lowest degree of embrittlement. This can be attributed to an appropriate polymer content, which enhances the low-temperature cracking resistance of asphalt at −10 °C. However, excessive polymer addition leads to an increase in its stiffness modulus, as observed in PU/HCRMA-2, resulting in reduced low-temperature cracking resistance at −10 °C.

### 3.10. Crosslinked Structure of PU/HCRMA

#### 3.10.1. FTIR Analysis

**Figure 11:**
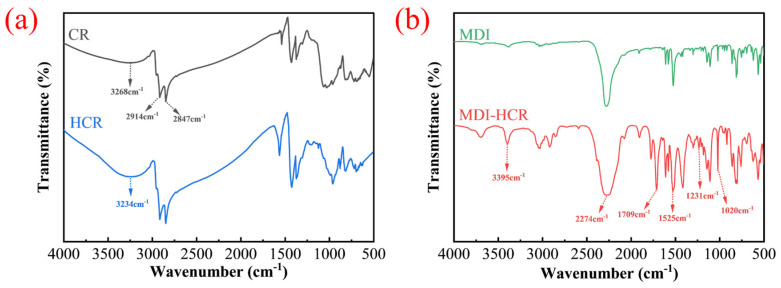
The results of FTIR tests. (**a**) The infrared spectra of CR and HCR. (**b**) The infrared spectra of MDI and MDI-HCR.

Figure 11a shows the infrared spectra of CR before and after modification. It can be observed that the rubber exhibits strong absorption peaks at 2914 cm^−1^ and 2847 cm^−1^, which are primarily attributed to the antisymmetric and symmetric stretching vibrations of C-H bonds. The broad peak in the range of 3150 to 3600 cm^−1^ corresponds to the O-H stretching vibration of hydroxyl groups [42]. Within the characteristic absorption region of hydroxyl groups, the O-H absorption intensity of HCR is significantly stronger than that of CR. This indicates that, under the strong oxidizing action of sodium hypochlorite, new hydroxyl groups are generated on the surface of CR. This is primarily due to the strong oxidizing capacity of sodium hypochlorite, which can cleave the C=C and C-H bonds in the surface rubber chains, leading to the generation of hydroxyl groups and other reactive oxygen-containing groups [26].

The infrared spectra of MDI before and after reaction with HCR are presented in Figure 11b. The results indicate that the absorption peak near 3395 cm^−1^ corresponds to the -NH group, while the peak at 2274 cm^−1^ represents the vibration of the NCO group. A peak also appears at 1709 cm^−1^, indicating the vibration of the C=O group. This was created by the consumption of NCO with an OH group to form urethane. The infrared spectra clearly show enhanced absorption peaks at 3395 cm^−1^, 1709 cm^−1^, 1525 cm^−1^, 1231 cm^−1^, and 1020 cm^−1^, which signify the formation of carbamate groups. Simultaneously, the hydroxyl group peak at 3234 cm^−1^ disappears. This demonstrates that MDI reacts with the hydroxyl groups on the surface of CR, resulting in the generation of carbamate groups. Through spectral comparison, it can be observed that the NCO group peak near 2274 cm^−1^ does not disappear after mixing HCR and MDI, indicating that not all isocyanate groups reacted with the HCR immediately after mixing. Furthermore, the isocyanate groups react with the active groups in the asphalt, such as hydroxyl groups, carboxyl groups, and amines [43]. Therefore, this study suggests that the isocyanate groups can react simultaneously with both asphalt and HCR, thereby forming a crosslinked structure. This crosslinked structure can be confirmed by conducting an FM test, as described in Section 3.10.2.

#### 3.10.2. Structure of PUMA and PU/HCRMA

**Figure 12:**
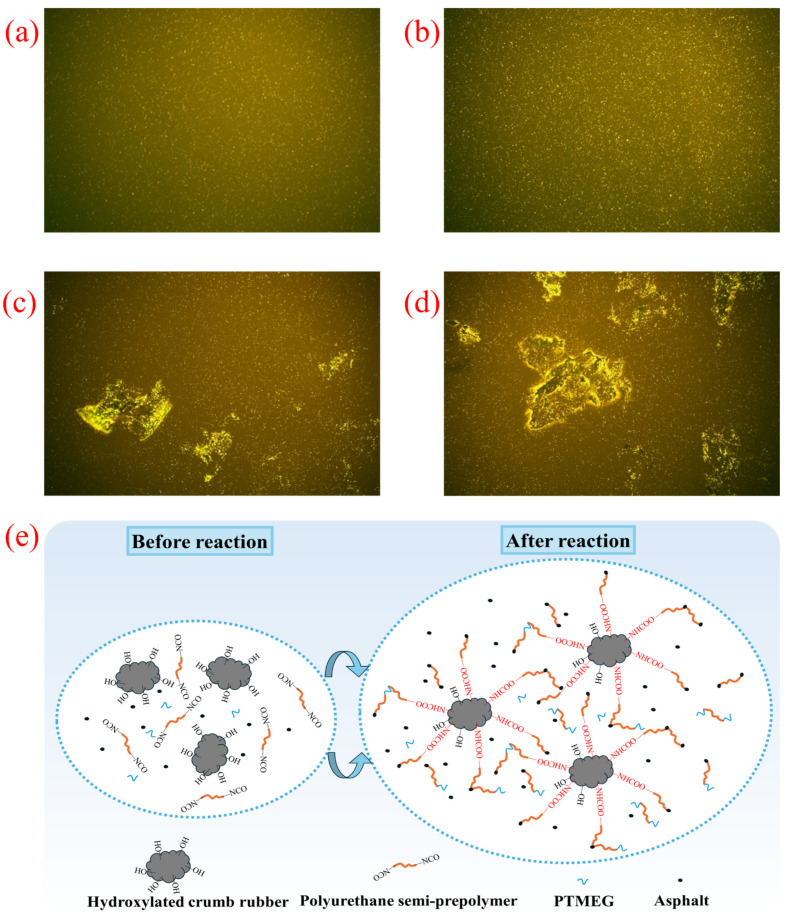
The results of FM tests and the modification mechanism. (**a**) PUMA-1. (**b**) PUMA-2. (**c**) PU/HCRMA-1. (**d**) PU/HCRMA-2. (**e**) The modification mechanism of PU/HCRMA.

FM utilizes the distinct fluorescent states of various substances under excitation to reveal their multiphase distribution. Figure 12a–d show the fluorescence micrographs of modified asphalt specimens. The yellow fluorescent regions represent the PU phase, while the dark areas correspond to the base asphalt and HCR. It can be observed that the PUMA prepared by the semi-prepolymer process exhibits good dispersion. Focusing on the vicinity of the HCR, it is noticeable that the fluorescence intensity on its surface is significantly higher than in other areas, indicating substantial accumulation of MDI on the HCR surface. Based on infrared spectroscopy and FM images, it can be inferred that the active groups in the polyurethane prepolymer continue to undergo chemical reactions with the HCR as the core, radiating outward to form a crosslinked structure, as schematically illustrated in Figure 12e. Due to the excellent elasticity of CR, it can serve as a load-absorption point, thereby imparting favorable deformation resistance and elastic recovery capability to the PU/HCRMA. The proposed mechanisms are supported by the results of the MSCR tests.

## 4. Conclusions

In this study, hydroxylated crumb rubber (HCR) was introduced to simultaneously enhance the rutting resistance and cracking resistance of polyurethane-modified asphalt (PUMA) while mitigating the adverse effects of crumb rubber (CR) on the storage stability of PUMA. A comprehensive performance evaluation was conducted on asphalt modified with PU and HCR (PU/HCRMA) and a PU/HCRMA asphalt mixture. The main findings are listed as follows:

(1) The formulation of PU/HCRMA was determined through an orthogonal design. With an isocyanate index of 2.5, a PU content of 6%, and an HCR content of 6%, PU/HCRMA exhibits excellent rutting resistance and cracking resistance compared to PUMA, with an improvement of 28.91% in the rutting factor at 64 °C and a reduction of 49.93 MPa in the stiffness modulus at −24 °C.
(2) The storage stability of PU/HCRMA has been improved. Incorporating HCR in PUMA enhances its viscosity and flow resistance while reducing temperature susceptibility. Furthermore, HCR endows PU/HCRMA with exceptional elastic recovery and deformation resistance. The PU/HCRMA asphalt mixture has demonstrated significant improvements in both rutting and cracking resistance compared to PUMA.
(3) Results from FTIR and FM confirm the reactions between active isocyanate groups and hydroxyl sites in HCR that form radially propagated crosslinked networks in PU/HCRMA. HCR functioned as the primary agent for stress storage and release, significantly improving the elastic recovery and viscoelastic stability of PU/HCRMA while reducing its energy dissipation.

Although this study conducted a series of performance investigations on polyurethane-modified asphalt through laboratory experiments, providing reliable solutions for its low-cost industrial application, the material has not yet been implemented in actual engineering projects due to time constraints. Further research is warranted to evaluate its long-term durability performance under aging and loading.

Furthermore, in practical applications, the paving process for PU/HCRMA mixtures is identical to that of conventional asphalt mixtures (e.g., SBS-modified asphalt mixtures), requiring no additional brand-new equipment. It is worth noting that the viscosity of PU/HCRMA increases over time until the isocyanate groups have completely reacted in the asphalt. The use of incompletely cured, low-viscosity PU/HCRMA in the preparation of asphalt mixtures will help reduce energy consumption.

## Figures and Tables

**Table 1:** The basic properties of raw materials for the preparation of PU.

Properties	Molecular Weight	Appearance	Purity (%)
MDI	250.250	White crystalline solid	>98
PTMEG	1000	White waxy solid	>98
DY-12	631.56	Colorless transparent liquid	>95

**Table 2:** The basic properties of CR.

Properties	Specifications	Test Result	Standard
Heating reduction (%)	≤1.0	0.24	GB/T 19208-2020 [24]
Ash (%)	≤10	9	
Acetone extract (%)	≤8	7	
Rubber hydrocarbon content (%)	≥45	58	
Carbon black content (%)	≥26	29	
Metal content (%)	≤0.05	0.02	
Relative density (10^3^ kg/m^3^)	≤1.20	1.198	
Sieve residue (%)	≤10	2.4	

**Table 3:** The basic properties of BA.

Properties	Test Values	Specifications	Standard
Penetration (25 °C, 0.1 mm)	72.9	60–80	JTG 3410-2025 [25]
Softening point (°C)	48.6	≥46	
Ductility (15 °C, cm)	161	≥100	
Density (g/cm^3^)	1.036	-	
Viscosity (135 °C, Pa∙s)	0.62	≤3	
Mass loss (%, After TFOT)	0.4	≤0.8	
Residual penetration ratio (25 °C, %, After TFOT)	70.6	≥61	
Residual ductility (15 °C, After TFOT)	27	≥15	

**Table 4:** Orthogonal experimental design with variations in isocyanate index (A), PU content (B) and HCR content (C).

Specimen Number	Isocyanate Index (A)	PU Content (wt%) (B)	HCR Content (wt%) (C)
1#	1.5	3	3
2#	1.5	6	9
3#	1.5	9	6
4#	2.5	3	9
5#	2.5	6	6
6#	2.5	9	3
7#	3.5	3	6
8#	3.5	6	3
9#	3.5	9	9

**Table 5:** The results of orthogonal experiments with variations in isocyanate index, PU content and HCR content.

Specimen Number	Penetration (25 °C, 0.1 mm)	Softening Point (°C)	Ductility (5 °C, cm)
1#	57.9	52.9	6.04
2#	51.9	61.4	5.63
3#	51.5	59.1	5.80
4#	49.2	58.2	6.53
5#	53.2	57.2	7.05
6#	56.2	55.1	6.66
7#	52.7	57.2	6.71
8#	53.6	54.6	7.13
9#	48.2	63.6	5.30

**Table 6:** The formulations of the selected modified asphalt types.

Number	Isocyanate Index (A)	PU Content (%) (B)	HCR Content (%) (C)
PU/HCRMA-1	2.5	6	3
PU/HCRMA-2	3.5	9	9
PUMA-1	2.5	6	-
PUMA-2	3.5	9	-

**Table 7:** The softening point difference test results for modified asphalt specimens.

Asphalt Specimens	Softening Point Difference (°C)
PU/HCRMA-1	0.7
PU/HCRMA-2	1.75
PUMA-1	1.1
PUMA-2	1.45

**Table 8:** The results of the Arrhenius equation fitting and flow activation energy.

Type	Fitting Equation	R^2^	Flow Activation Energy (kJ/mol)
PU/HCRMA-1	y = 11,262.36x − 26.71	0.99917	93.64
PU/HCRMA-2	y = 11,402.11x − 26.73	0.99722	94.80
PUMA-1	y = 11,023.50x − 26.62	0.99967	91.65
PUMA-2	y = 11,070.30x − 26.47	0.99882	92.04
BA	y = 9974.02x − 24.88	0.99782	82.92

## Data Availability

The original contributions presented in this study are included in the article. Further inquiries can be directed to the corresponding author.

## Abbreviations

The abbreviations used in this research are defined as follows:

**Abbreviation**	**Definition**
BA	Base asphalt
BBR	Bending beam rheometer
CR	Crumb rubber
DSR	Dynamic shear rheometer
DY-12	Dibutyltin dilaurate
FM	Fluorescence microscopy
FTIR	Fourier transform infrared spectroscopy
HCR	Hydroxylated crumb rubber
MDI	4,4′-Methylene diphenylmethane diisocyanate
MSCR	Multiple-stress creep recovery
PTMEG-1000	Poly (tetramethylene ether glycol) 1000
PU	Polyurethane
PUMA	Polyurethane-modified asphalt
PUSP	Polyurethane semi-prepolymer
PU/HCRMA	Polyurethane and hydroxylated crumb rubber composite-modified asphalt

## References

[1] Yu J., Chen F., Deng W., Ma Y., Yu H. (2020). Design and performance of high-toughness ultra-thin friction course in south China. Constr. Build. Mater..

[2] Zhao S., Ouyang J., Zhou L. (2025). Rheological Properties of Polyurethane-Modified Asphalt and Its Effect on the Performance of Porous Asphalt Mixes. J. Mater. Civ. Eng..

[3] Lu J., Xu S., Li C., Fu C., Gao M., Wang Z., Yang F., Zhou G., Li R., Ling T. (2025). The latest research progress of polyurethane modified asphalt binder: Synthesis, characterization, and applications. Int. J. Adhes. Adhes..

[4] Lyu L., Wang Z., Ji J., Li Y., Wen Y., Zhang J., Li R., Chen Z., Pei J. (2022). Investigating rheological and healing properties of asphalt binder modified by disulfide-crosslinked poly(urea-urethane) elastomer. Constr. Build. Mater..

[5] Jiang W., Yuan D., Zhang S., Bao R., Xiao J., Wu W., Wang T. (2023). Experimental analysis of deformation-adapted binders and their mixture performance. Constr. Build. Mater..

[6] Bartolomé L., Aurrekoetxea J., Urchegui M.A., Tato W. (2013). The influences of deformation state and experimental conditions on inelastic behaviour of an extruded thermoplastic polyurethane elastomer. Mater. Des..

[7] Zhang Y., Zhao X. (2026). Disulfide-Crosslinked Polyurethane-Modified Asphalt: Balancing Fatigue Resistance and Healing Through Dynamic Covalent Networks. Polymers.

[8] Han B., Xing Y., Li C. (2025). Investigation on Dynamic and Static Modulus and Creep of Bio-Based Polyurethane-Modified Asphalt Mixture. Polymers.

[9] Khair A., Wang L., Li H., Han Y., Lin Z., Sun Y., Zhang H. (2026). Comparative performance evaluation of asphalt binder modified with high-content pretreated crumb rubber and various additives. J. Road Eng..

[10] Wu M., Li M., Yin L., You Z. (2026). Asphalt-rubber interaction in crumb rubber modified asphalt: A review. J. Clean. Prod..

[11] Ji W., Liang S., Xu W. (2026). Crumb rubber modified straw oil-based asphalt (CR-SOBA): A multi-scale investigation linking molecular interaction to macroscopic performance. Mater. Today Commun..

[12] Zhu H., Zhang M., Li Y., Zou Y., Chen A., Wang F., Liu L., Gu D., Zhou S. (2022). Swelled Mechanism of Crumb Rubber and Technical Properties of Crumb Rubber Modified Bitumen. Materials.

[13] Kök B.V., Yilmaz M., Geçkil A. (2013). Evaluation of Low-Temperature and Elastic Properties of Crumb Rubber– and SBS-Modified Bitumen and Mixtures. J. Mater. Civ. Eng..

[14] Dong D., Huang X., Li X., Zhang L. (2012). Swelling process of rubber in asphalt and its effect on the structure and properties of rubber and asphalt. Constr. Build. Mater..

[15] Wu M., Boateng K.A., Yin L., Liu Z., You Z., Jin D. (2025). High-content crumb rubber modified asphalt mixture via wet process: Laboratory evaluation and field application. Constr. Build. Mater..

[16] Li J., Xiao F., Amirkhanian S.N. (2020). High temperature rheological characteristics of plasma-treated crumb rubber modified binders. Constr. Build. Mater..

[17] Yang X., Shen A., Li B., Wu H., Lyu Z., Wang H., Lyu Z. (2020). Effect of microwave-activated crumb rubber on reaction mechanism, rheological properties, thermal stability, and released volatiles of asphalt binder. J. Clean. Prod..

[18] Kocevski S., Yagneswaran S., Xiao F., Punith V.S., Smith D.W., Amirkhanian S. (2012). Surface modified ground rubber tire by grafting acrylic acid for paving applications. Constr. Build. Mater..

[19] Tian N., Tataranni P., He Y., Sangiorgi C. (2025). Surface treatment of waste tire rubber via oxidation and alkalization for enhanced compatibility with bitumen. Constr. Build. Mater..

[20] Yang X., Li S., Dou H., Yang W., Jia X., Dai J., Liang J. (2025). Study on properties and reaction mechanism of waste cooking oil-microwave activated crumb rubber/PPA composite modified asphalt. Constr. Build. Mater..

[21] Li J., Cao W., Fan X., Zhang J., Li Y., Wang S., Meng F. (2026). Effects of crosslinking density on properties of crumb rubber/SBS composite modified asphalt: Insights into microstructure evolution and property correlation. Constr. Build. Mater..

[22] Zhang H., Gong M., Gao D., Yang T., Huang Y. (2020). Comparative analysis of mechanical behavior of composite modified asphalt mixture based on PG technology. Constr. Build. Mater..

[23] Zhang Z., Liu H., Zhu Y., Chen L., Sun J., Wang L., Huang T. (2025). Modification of bitumen with polyether-based polyurethanes containing different hard segments. J. Traffic Transp. Eng.-Engl. Ed..

[24] (2020). Ground Vulcanized Rubber.

[25] (2025). Standard Test Methods of Asphalt and Asphalt Mixture for Highway Engineering.

[26] Han L., Zheng M., Li J., Li Y., Zhu Y., Ma Q. (2017). Effect of nano silica and pretreated rubber on the properties of terminal blend crumb rubber modified asphalt. Constr. Build. Mater..

[27] Yang F., Gong H., Cong L., Shi J., Guo G., Mei Z. (2022). Investigating on polymerization process and interaction mechanism of thermosetting polyurethane modified asphalt. Constr. Build. Mater..

[28] Yang T., He Z., Huang G., Zhao Y., Fu J., Xiang H., Zhou Y. (2023). Study on materials composition and process parameters of polyurethane-modified asphalt synthesized in-situ by the one-shot process. Constr. Build. Mater..

[29] Zhou T., Liu R., Cao L., Jin C., Dong Z., Gershome A.G., Huang Q. (2025). Integration of warm mix technology and high crumb rubber content for enhanced asphalt treated base material. J. Clean. Prod..

[30] Kataware A.V., Darshan N., Singh D. (2025). Utilisation of activation energy determined from viscosity to assess the rutting performance of asphalt binders containing various warm mix additives. Constr. Build. Mater..

[31] Xu B., Blok R., Teuffel P. (2023). An investigation of the effect of relative humidity on viscoelastic properties of flax fiber reinforced polymer by fractional-order viscoelastic model. Compos. Commun..

[32] Olard F., Di Benedetto H. (2003). General “2S2P1D” Model and Relation Between the Linear Viscoelastic Behaviours of Bituminous Binders and Mixes. Road Mater. Pavement Des..

[33] Jiang W., Zhang M., Ren P., Xing C., Yuan D., Wu W. (2024). Development of porous asphalt mixture based on the synthesis of PTEMG and MDI polyurethane asphalt. Constr. Build. Mater..

[34] Liu H., Zhang Z., Zhu Y., Sun J., Wang L., Huang T., Chen L. (2022). Modification of asphalt using polyurethanes synthesized with different isocyanates. Constr. Build. Mater..

[35] Cong P., Xun P., Xing M., Chen S. (2013). Investigation of asphalt binder containing various crumb rubbers and asphalts. Constr. Build. Mater..

[36] Li F., Yang Y. (2020). Understanding the temperature and loading frequency effects on physicochemical interaction ability between mineral filler and asphalt binder using molecular dynamic simulation and rheological experiments. Constr. Build. Mater..

[37] Acharjee P.K., Souliman M.I., Khalifah R., Elwardany M. (2024). Frequency- and temperature-dependent dynamic shear modulus and phase angle prediction models based on existing asphalt binder viscosity data using Artificial Neural Network (ANN). Constr. Build. Mater..

[38] Ding H., Gong H., Cong L., Hou Y., E G. (2024). Investigating influence of hard segment content on rheological behavior of thermosetting PU modified asphalt. Constr. Build. Mater..

[39] Liu H., Zhang Z., Zhang S., Chang P., Liang Y., Wang Z., Ban X., Guo Y., Liu X. (2024). Study on the effect of soft segment length on the performance of polyether-based polyurethane modified asphalt. Int. J. Adhes. Adhes..

[40] Yan W., Ou Y., Xie J., Huang T., Peng X. (2021). Study on Properties of Bone Glue/Polyurethane Composite Modified Asphalt and Its Mixture. Materials.

[41] Zhuang W., Ren S., Liu B., Ding T., Liu L., Gu L., Sun M. (2025). Modification Mechanism and Performance of High-Content Polyurethane-Modified Asphalt. Coatings.

[42] Phiri M.M., Phiri M.J., Formela K., Hlangothi S.P. (2021). Chemical surface etching methods for ground tire rubber as sustainable approach for environmentally-friendly composites development—A review. Compos. Pt. B-Eng..

[43] Gong X., Liu Q., Chen P., Wang H., Liu X., Chen S., Wu S. (2024). Modification mechanism of green polyurethane modified asphalt prepared by in-situ polymerization. Constr. Build. Mater..

